# Working Memory Capacity, Inhibitory Control and the Role of L2 Proficiency in Aging L1 Dutch Speakers of Near-Native L2 English

**DOI:** 10.3390/brainsci3031261

**Published:** 2013-08-19

**Authors:** Merel Keijzer

**Affiliations:** Applied Linguistics, Faculty of Arts, University of Groningen, Oude Kijk in ‘t Jatstraat 26, 9712 EK Groningen, The Netherlands; E-Mail: m.c.j.keijzer@rug.nl; Tel.: +31-50-363-7273; Fax: +31-50-363-5821.

**Keywords:** working memory capacity, inhibitory control, near-nativeness, L2 proficiency, aging

## Abstract

This paper examines the intricate relationship between working memory (WM) capacity and inhibitory control as a function of both L2 proficiency and age. In both its design and research questions, this study closely follows Gass & Lee’s work, where both L1 and L2 Reading Span Tasks (as measures of WM capacity) and L1 and L2 Stroop interference tasks (to measure inhibitory control) were administered. In this study, the test battery is augmented by both an L1 and L2 C-test of overall language proficiency. Participants were 63 L1 Dutch speakers of L2 English, who had been immersed in an L2 environment for a considerable amount of time. Their data were set off against those of 54 monolingual Dutch speakers and 56 monolingual English speakers. At the time of testing, all the bilingual participants had a near-native command of English and their L1 and L2 WM scores were not found to be significantly different. However, discrepancies did occur in Stroop test scores of inhibition, where the bilinguals performed better in their L2 English than L1 Dutch. These main effects often contradicted the results found in Gass & Lee’s study, who examined less proficient L2 learners. An aging effect was furthermore found: older subjects consistently performed more poorly on WM and inhibition tasks than their younger peers. These results can shed light on how individual factors like WM capacity and inhibitory control interact in successful late bilinguals and how these dynamics shift with advanced age.

## 1. Introduction

For a long time, the success of second language (L2) acquisition was thought to mainly depend on the age of acquisition; building on the premises of the Critical Period Hypothesis [1], young children were considered to have an advantage in ultimate attainment compared to those who acquired their second language at a post-puberty age. Although this “the earlier the better effect” has generally been found to be robust, there are notable exceptions of late second language learners who nonetheless attain near-native or native levels of L2 proficiency, on lexical, phonological, morphological, syntactic, semantic, or all of these aspects [2]. Such exceptions have typically been explained in the light of individual differences concerning motivation, language learning setting (naturalistic *versus* classroom), and language aptitude [3]. The precise nature of language aptitude, however, remained rather vague until the claim, relatively recently, that language aptitude mainly builds on working memory capacity and, more broadly, executive control comprising such constructs as inhibitory and attentional control [4]. Cognitive psychology studies have earlier shown that both working memory capacity and executive control can fluctuate dramatically across individuals on the basis of socio-economic background, but also age (maturing in children and declining in older adults) [5].

“Learning a second language involves sorting through a great deal of lexical, phonological, morphological, and syntactic information to determine what is relevant and what is not” [6]. Because of that, it is perhaps counterintuitive that, apart from notable exceptions ([7], very few studies have examined the role that inhibitory control plays in L2 success. At the same time, a better developed working memory (WM) allows learners to hold more and longer strings of linguistic information in mind while processing [8] and it is therefore not surprising that recent studies have also considered WM capacity in relation to L2 ultimate attainment. However, the interaction between both these constructs has not received much attention. There is one notable exception to this: Gass & Lee [6], who examine the relationship between WM capacity and inhibitory control in two groups of L2 learners of Spanish (L1 American English): one beginner, and one more advanced group of learners. They are particularly interested in how L2 proficiency may predict L2 control. In comparing scores obtained from an L1 WM test to L2 WM scores, it was established that the correlation between both scores was significant for more advanced learners, but not for the beginning L2 learners. L2 proficiency was not found to influence inhibitory control. 

While [6] fills a pre-existing gap in L2 acquisition research, the authors acknowledge that no objective measure of L2 proficiency was used (proficiency was instead operationalized as the number of years of Spanish classes). Perhaps most importantly, even the advanced learners had a limited command of Spanish and the authors therefore argue that a re-examination of this issue in highly fluent L2 speakers is needed, especially with respect to inhibitory control, as in the design in [6] “both groups of learners seemed to be able to bypass language when doing the Stroop test” (2011: 79) and future work needs to establish if more Stroop interference is found with increased L2 proficiency levels, where language processing becomes an integral task component. 

The current study sets out to fill that niche: it examines 63 L1 Dutch speakers of L2 English who moved to their L2 environment (Australia) at a mean age of 27.23 (SD 9.734; range 13–61 years). Although some had received minimal English instruction at school while still residing in their L1 Dutch environment (The Netherlands), most had not. In all cases, the bulk of L2 acquisition took place in an immersion, naturalistic setting, making the participants late learners. An objective C-test of general L2 language proficiency was administered to measure participants’ language proficiency and revealed all speakers to have an near-native command of their L2 English to the extent that they could be considered bilingual (see Section 3 below). The C-test is a variant of a cloze test, where parts of words rather than whole words are deleted and several smaller texts are administered rather than one lengthier text, which is common practice in cloze formats. The data of the bilingual group was set off against that of a monolingual Dutch group (*n* = 54) and a monolingual (Australian) English group (*n* = 56) (see Section 3 below for more details). Although the focus of this study, following that of [6], is very much on the interaction of WM capacity and inhibitory control in relation to L2 proficiency, the participants in this study are not college-aged adults, as in [6]. Instead, they fall in one of three age categories: a middle-aged 40–50 group, a “youngest-old” 60–70 group, and an “oldest old” 71+ group. This adds an aging dimension to the data, which can be insightful in an examination of the interaction between WM capacity and inhibitory control, as both constructs are well-known to diminish with advanced age [8].

## 2. Working Memory Capacity and Inhibitory Control across the Lifespan

### 2.1. Working Memory Capacity

In its most rudimentary form, WM can been described as involving “the temporary storage and manipulation of information that is assumed to be necessary for a wide range of complex cognitive activities” [8], among which language. Over the years, an array of WM models have been proposed, mostly modifications to and elaborations on the model proposed by Baddeley & Hitch [9]. In this model two so-called slave systems work in parallel: the phonological loop (responsible for maintaining and rehearsing linguistic information) and the visuospatial sketchpad (for storing visual and spatial information). These two slave systems are overseen by a central executive, a system that coordinates the division of attention and ensures that irrelevant information is ignored, especially in complex tasks [8,10]. In the late 1990s, this model was augmented by an episodic buffer, taking over the storage function previously ascribed to the executive control system. This left the central executive entirely as an “attentionally-based control system” [10]. 

While it is deliberately not the intention of this paper to elaborate on either the originally proposed WM model or modifications of it, it is essential to recognize WM overall, and the central executive in particular, as a limited capacity system. Information processing and storage functions work simultaneously [11] and there is constant competition between the two, leading to a trade-off situation in which WM resources are either primarily allocated to processing or storage. If processing is more efficient and faster, more storage space is available. In a beginning L2 learner, virtually all resources need to be allocated to processing. As L2 proficiency increases, processing will become more automatic, resulting in more available storage for, for instance, L2 words [6]. 

It is well established that individuals differ in their WM capacity, which has been accounted for by differences in general cognitive resources, attention span, *etc.* [12,13], but can, perhaps, all be reduced to Daneman & Carpenter’s [14] argumentation that the computations that have to be achieved using WM do not differ across individuals, but the speed and efficiency with which individuals carry out these computations do. Perhaps the best way to test individual WM capacity is by providing participants with a demanding processing task, where both the processing and storage components of WM are taxed at the same time. Commonly administered WM tests like digit or word spans do not suffice as they are only compatible with the storage component. Better, it has been argued, is a format like the Reading Span Task (developed by [14]), which is compatible with WM characteristic as they have been charted over the past years: the test projects sentences, one-by-one, for a brief period of time, the time it takes to read the sentences out loud at a normal pace. Test takers are instructed to read the sentences out loud, paying close attention to their content, as they are quizzed for comprehension afterwards. In addition, they are asked to memorize the final word of each sentence and are subsequently asked to recall these words at regular intervals. More efficient readers are expected to recall more words [14], although what makes someone an efficient reader is left unspecified. Experience is a likely factor and, related to that, years of formal education, as higher education typically depends on reading many (varied) texts. 

The limited capacity nature of the WM system is most evident in young children and older adults due to limited resources in both populations. Focusing on older adults, cognitive changes are noticeable in this group due to biological and chemical changes in the neutral substrate, and this is party manifested in reduced WM capacity [15,16]. In fact, this reduction in WM capacity itself is often held responsible for other age-related deficits in numerous cognitive tasks, including syntactic processing and problem solving tasks [8]. The question then becomes whether the age-related WM deficits are processing or storage-related and no consensus has been reached there. Several studies have suggested that it is mainly the processing component that causes the changes [17], sometimes more broadly construed as an overall reduction in processing speed [18]. This argument is underscored by the finding in [19] that an interaction exists between age and sentence complexity on a Reading Span Task, but not between age and mnemonic load, suggesting that it is not so much the ability to keep items in memory, but the ability to process information that is impaired. Other studies, however, have not replicated this interaction, instead ascribing age-related WM impairments to the storage capacity of WM [20]. In [21], also, no differences were observed in how fast (reaction times) younger and older subjects processed new words, but were found in recall performance (with elderly participants recalling far fewer items). There appears to be a general consensus that elderly subjects show more of a trade-off, or competition, between the processing and storage components of working memory. In other words, the already limited capacity systems is even more taxed and this should be clearly revealed on the basis of a complex WM test like the Reading Span Task.

When elderly subjects are, in addition, bilingual, the picture becomes even more complex. A much-debated issue is whether the language in which a Reading Span Task is administered influences the results. Although results are not always uniform, it would appear that L1 and L2 Reading Span Test scores approximate each other more in high-proficient L2 speakers [6]. Beginning L2 learners’ L1 and L2 Reading Span Test scores, by contrast, tend to deviate more [22,23]. 

### 2.2. Inhibitory Control

The basic description of inhibitory control is the ability to focus on one thing while ignoring irrelevant or no longer relevant competing information. An illustrative linguistic example is the ability to select the correct word in the face of many competitors that are semantically and/or phonologically related. In bilingual situations, not only same-language competitors have to be suppressed, but inhibition also reflects the ability to block the language currently not in use. 

Just as there are essentially two constructs underlying WM capacity (processing and storage), inhibition too is characterized by an interplay between inhibition on the one hand and successful activation of the target on the other. Indeed, activation and inhibition are very much two sides of the same coin. Also similar to WM capacity, numerous models have been proposed to capture this dichotomy; one of the best-known is perhaps the Inhibitory Control (IC) model by Green [24]. In a set-up not unlike Baddeley’s slave systems and executive control model, Green proposes a coordinating Supervisory Attentional System (SAS) that not only oversees that the appropriate schemas are activated, depending on the message that the speaker intends to convey, but also—in bilingual situations—ensures that these schemas are activated in the target language. In later years, this model was complemented by the also commonly cited Activation Threshold Model by Paradis [25], in which the constructs frequency and recency of use were introduced: the more often and recently used language (and items within that language, which can be whole words, but also smaller units like phonemes) has a lower threshold than the language that is used less recently and frequently. Recent and frequent use of a language thus facilitates subsequent activation. 

While it is deliberately not the intention of this paper to discuss the intricacies of each model, it is important to note that inhibition is commonly seem as one of the basic components of executive functioning (abbreviated as EF), also referred to as executive control, which in turn denotes “a multicomponent construct that consists of a range of processes that are involved in the planning, organization, coordination, implementation, and evaluation of our non-routine activities” [8]. 

The past decade has seen an increased focus on executive control and it is now generally accepted that executive control varies widely among individuals. The cause of this variation has been attributed to a range of factors and mechanisms, “biological, psychological, health-related, environmental, and lifestyle” [8]. Individual differences in inhibitory control have been assessed by means of many different paradigms, ranging from card sorting tests, go-no-go and stop-signal tests, Simon tests, Flanker tests, and—also very commonly employed—Stroop designs. In this latter test, participants are asked to respond to the color of the ink in which symbols (mostly words) are written, suppressing the actual names of the colors. For example, the word “black” may be written in yellow ink and participants are asked to respond to yellow, ignoring black. Thus, trials can either be congruent (the ink color and word correspond) or incongruent (where there is a discrepancy between the two) and the so-called Stroop effect is computed by subtracting the reaction times on the congruent trials from those on the incongruent trials (see [6] for a methodological discussion). One of the most robust findings of the Stroop test is that skilled readers show longer response latencies and make more errors on the incongruent trials than the congruent trials, reflecting their automatic processing of the written word that has to be inhibited in the Stroop test [6]. 

The Stroop test has also commonly been employed in L2 acquisition research and bilingual situations, but the results here are less uniform. Generally, advanced L2 speakers experience more interference effects in both their languages, in contrast to beginning L2 learners, who have less difficulty with Stroop tests administered in their weaker L2, as no language processing is necessarily involved here. However, in [6], comparing beginning and more advanced L2 learners of Spanish, the results for both groups were indicative of very little language processing. Results of studies looking at the interaction between L2 proficiency and L2 inhibition scores have often been interpreted in the light lexical access models and fit in particularly well with the Revised Hierarchical Model ([26]; for an update, see [27]), which proposes that beginning L2 learners access words via their L1 (word association), while more proficient L2 users directly access the meaning of words via their L2 (concept mediation). 

The individual variation that is found for Stroop-like paradigms is also partly age-related; executive control improves during childhood and declines in older adults [28], due to neurochemical changes in the prefrontal cortex, on which inhibition crucially depends. That is not to say that all elderly subjects necessarily show impaired inhibition and “some older adults retain excellent cognitive function well into their 70s and 80s, and perform as well as or better than younger adults” [8], but neuroimaging studies have been able to reveal a compensatory mechanism in these older adults even when they do not differ from their younger peers on behavioral measures like the Stroop: older adults often display more and more diffuse brain activation in carrying out the same tasks than younger adults [16]. Proficient bilingual speakers have even been found to be able to attenuate the usual age-related decline in inhibitory control due to enhanced cognitive control they gained through a lifelong need to activate and inhibit several language systems (*cf.* [29]). 

### 2.3. The Interaction between Working Memory Capacity and Inhibitory Control

The discussion above indicates that WM capacity and inhibitory control cannot always be teased apart easily. In fact, it is even unclear whether they tap into different constructs. Perhaps this has been most discussed in an aging context. Hasher, Zacks, and May [30], for instance, attribute age deficits in WM capacity to a failure to inhibit information that is no longer relevant. This is evidenced, for instance, in participants performing a Reading Span Task and naming words belonging to previous trials. It should therefore come as no surprise that past studies have found both WM capacity and inhibitory control to be highly dependent on each other.

Kane & Engle [31], in particular, assessed both WM capacity (through an operation span test) and inhibitory control (through a series of Stroop tests) and found that individuals with a high WM score consistently produced better inhibition scores than individuals who had scored lower on the WM task. This has been taken to suggest that “attentional control for native speakers of a language can be predicted by differences in working memory capacity” [6]. In an attempt to see whether the same effect was found for L2 learners, [6] conducted a Reading Span Test in both the L1 and L2 of L1 English learners of L2 Spanish of varying proficiency levels. While no significant correlation was found between L1 WM scores and L2 inhibition scores for the lower-level students, the more proficient ones did show such a significant correlation. In other words, “with increased proficiency, the relationship shows a pattern similar to that if monolinguals” [6]. It remains an empirical question whether this relationship can also be established for much more advanced L2 speakers and how aging impacts on the results. 

### 2.4. Research Questions and Predictions

The current study elaborates on [6] as the first study to relate both WM capacity and inhibitory control to L2 proficiency. As such, and based on the theoretical discussion above, the research questions underlying this study also closely resemble those formulated in [6], with the additional factor of age. 

#### 2.4.1. Working Memory Capacity

1 Does L2 proficiency relate to L2 Reading Span Task scores? Does this relationship fluctuate as a function of age? Predictions: previous work has found that, with increased L2 proficiency, L1 and L2 Reading Span Scores more closely approximate each other. Given the high-proficient L2 speakers under investigation, who are in fact more accurately termed bilinguals, the majority of subjects will likely show a link between L1 and L2 WM scores. The effect is furthermore expected to remain the same across all age groups, including older adults, but their score on the Reading Span Test in both languages is expected to be lower due to age-related lexical access difficulties (see [15]).
2 Can L2 WM scores (as measured by a Reading Span Task) be predicted on the basis of L1 WM scores? Does this depend on L2 proficiency and/or age of the test taker? Predictions: similar to the previous prediction, L2 WM scores can be better predicted on the basis of L1 WM scores in high-proficient L2 speakers, encompassing the vast majority of participants in this study. As both L1 and L2 WM scores are likely to decline as a function of advanced age rather than merely one language, this effect should remain intact, also in the older adults.

#### 2.4.2. Inhibitory Control

3 Can language proficiency be related to inhibiting interfering linguistic information (as measured by a Stroop task)? Can differential effects be found for the L1 and L2? And is this effect the same for younger and older adults? Predictions: because of the bilingual participants under investigation, no large discrepancies are expected between L1 Dutch and L2 English Stroop test performance; the language knowledge in both languages will be sufficient for interference to play a role. Having said that, it is likely that more interference occurs in the Dutch version, given that Dutch likely remains the dominant language of the participants. However, the largest Stroop effects are expected for older adults, as these people tend to have more difficulty inhibiting irrelevant information. In other words, the response latencies are expected to be longest in the oldest participants, due to reduced inhibitory control (see Section 2.2) This is likely to be felt for both congruent and incongruent trials as well as the discrepancy between both. The accuracy scores are expected to follow the trends of the RTs: older people will likely obtain lower accuracy scores, and lower accuracy scores may be predicted for the most dominant language, *i.e.*, Dutch.

#### 2.4.3. Interaction Working Memory Capacity and Inhibitory Control

4 Does WM capacity relate to inhibitory control of linguistic information? Furthermore, can a relationship be established between L1 WM capacity and the ability to inhibit linguistic information in the L2? Does this relationship depend on age? Predictions: those individuals with higher WM capacity (better scores on the RST) will perform better on the Stroop test. In line with previous work, an inverse relationship is expected between L1 WM scores and L1 Stoop scores (a better performance is signaled by a smaller Stroop effect). Furthermore, L2 WM scores and L2 Stroop task scores are also likely linked in a similar way, as are L1 WM scores and L2 Stroop test scores, because the L2 and L1 processing is not expected to be substantially different in the bilingual group in this study. No differential effect is expected for the older adults, as the scores on both the Stroop and Reading Span Tasks are likely to decrease exponentially.

## 3. The Study

### 3.1. Participants

Participants in this study were 63 L1 Dutch speakers who were born and raised in The Netherlands. None had been raised bilingually. At a mean age of 27.23 (SD 9.734; range 13–61 years) they moved to an English (Australian) environment, where they subsequently learned English as their L2 in a naturalistic setting, although a number of participants indicated that they had had basic English classes at school prior to immigration. To look more closely at bilingualism effects, the data of these participants were compared with those of 54 Dutch monolinguals and 57 (Australian) English monolinguals, all of whom had never lived abroad for a substantial period of time. It must be pointed out that, although the English native speakers were true monolinguals, Dutch native speakers in general invariably have at least a rudimentary and receptive command of one or several foreign languages. It is furthermore important to note that the monolingual data were purely used for control purposes, with the focus very much being on the bilingual group. As such, the monolingual data is not reported in the tables. The participants were divided into three groups on the basis of their age: a middle-aged group between 40 and 50 years old and two older adult groups: one “youngest” old (60–70 years) and one “oldest old” (71+ years). Table 1 below lists several participant demographics, split per age group: the participants’ mean ages, their male/female ratio, years of formal education, and how long they had resided in the L2 environment at the time of testing. 

**Table 1:** Demographic information of participants, split per age category.

Group	Mean age	Female/Male ratio	Years of formal education	Length of residence (in years)	Age of L2 acquisition *
40–50 (*n* = 17)	43.12 ± 2.395	11 female 6 male	19 ± 1.936 Range: 15–24	9.12 ± 8.753 Range: 1–30	34.06 ± 8.67
60–70 (*n* = 17)	64.00 ± 3.873	12 female 5 male	16.29 ± 3.443 Range: 9–20	36.94 ± 8.555 Range: 25–50	27.12 ± 7.63
71+ (*n* = 29)	77.93 ± 4.734	13 female 16 male	12.89 ± 3.304 Range: 7–20	54.81 ± 7.321 Range: 25–61	22.85 ± 9.30

* It needs to be pointed out that age of L2 acquisition here is equated with age at emigration; in other words, it is the start of naturalistic L2 acquisition. This picture is in a way distorted because the youngest subjects typically had had much more formal English instruction at school while still residing in The Netherlands than their older peers.

Employing one-way ANOVA tests of variance for the independent variables of years of formal education, length of residence and age of L2 acquisition yielded significant differences between the age groups, starting with years of education (*F*(2,59) = 22.187, *p* < 0.000). Follow-up Tukey procedures revealed that all groups differed significantly from one another in a stepwise manner: the middle-aged group had received more schooling than the youngest old group (*p* < 0.05) but also when compared to the oldest group (*p* < 0.000). The participants aged 60–70 in turn went to school longer than their oldest old peers (*p* < 0.005). Similarly, the length of residence differed, perhaps unsurprisingly, across age groups: *F*(2,57) = 163.666, *p* < 0.000; *p* < 0.000 for all ages. The 40–50 year-olds had been in Australia for a shorter period of time than their 60–70 year-old counterparts, who in turn were outstayed by the oldest subjects. Finally, the onset of naturalistic L2 acquisition was found to be significantly different across age groups: *F*(2,57) = 8.571, *p* < 0.001. Upon closer inspection, it was the youngest group that had started their L2 immersion significantly later than the 71+ group (*p* < 0.000). While the middle-agers were not found to be significantly different from the “youngest old”, the discrepancy scores did border significance levels (*p* = 0.060). 

### 3.2. Materials and Procedure

A test battery, lasting approximately 2.5 h, was administered individually for each participant. During this time participants were asked to complete a variety of language tasks and neuropsychological measures. Many of these tasks were administered in both their L1 (Dutch) and L2 (English). The languages were counterbalanced: the participants were first of all presented with the Dutch stimuli before the English stimuli were presented. A 15 to 30 min break marked the transition from one language to the next. All instruction took place in the language of administration (Dutch for the first part of the test battery, and English for the second). All testing took place in a quiet room at Monash University in Melbourne, Australia or, in a few cases where participants did not have any means of transportation, in a quiet room in participants’ homes. The test sessions were mainly computer-based, but also included an orally administered language and social background questionnaire. Participants received a 30 Australian-dollar reimbursement for their participation (20 Australian dollars and 20 euros for both monolingual groups, respectively). For the purpose of this paper, the focus is solely on three of the paradigms that were included in the test battery: C-test, Reading Span Task, and Stroop Task. 

#### 3.2.1. C-Test of Proficiency

The C-test is a variant of the cloze procedure (a text with gaps that have to be completed by the test taker), which became very popular in foreign language testing in the 1980s due to its easy construction and scoring method [32]. Cloze procedures are generally believed to tap into lower-level overall language proficiency, such as knowledge of vocabulary, grammar, and idioms. Cloze formats build on the concept of reduced redundancy and internalized pragmatic expectancy grammar developed by Oller [33]. Any “reduction of redundancy” in a text, which is created through the deletion of words, will increase the processing difficulty for non-native speakers, while no strong effects are expected for native speakers, who will expect certain words and word classes in a given position (hence the expectancy grammar; [34]). Like the cloze test, C-tests also build on reduced redundancy and expectancy grammar, but unlike the cloze paradigm, C-tests consist of several smaller texts in which parts of words are deleted as opposed to whole words [35]. Two C-tests were employed in this study (one to assess L1 Dutch proficiency and one to do so for L2 English). Both versions were administered in the bilingual group, while the monolinguals were only asked to complete the task in their native language. Each C-test consisted of five texts that each contained 20 gapped items. Both texts had been standardized prior to testing according to the guidelines set out in [36].

Participants were given a maximum of 5 min per text, the rationale being that time pressure can distinguish between individual levels of expectancy grammars. All blanks and unacceptable words (with respect to grammar and/or text content) were considered incorrect (and awarded a score of 0), while all intended words or acceptable alternatives were scored as correct (a score of 1). Acceptable alternatives belonged to the same word class as the original word and also semantically fitted the context. Correct alternatives or original words containing spelling errors were also considered correct. With a total of 20 gaps per text, the maximally obtainable score on both the L1 Dutch and L2 English C-test was 100, allowing for easy comparison between the two languages. 

#### 3.2.2. Reading Span Task

The standardized Reading Span Tasks (L1 Dutch and L2 English) used in this study were short forms (60 rather than 100 sentences) of those developed by van den Noort *et al.* [37]. As part of the procedure, subjects were presented with sentences they were asked to read out loud but also scan for content. They were additionally asked to remember each sentence-final word. In [37], the tests to control for what they considered flaws of Reading Span Tasks employed until then were developed: previous studies controlled for number of words per sentence, but [37] also controlled for number of syllables and letters; the length, frequency, and abstractness/concreteness of the sentence-final words were also controlled for; in terms of measurement, not the longest set of sentences for which participants could recall the final word, but the entire number of sentence-final words recalled constituted the total Reading Span score. 

Whereas [37] used E-prime software to program the Reading Span Test, the version used in this study was constructed using Zep experimental software (developed by Utrecht University in The Netherlands), run under Linux Ubuntu. Sitting approximately 60 cm from a white laptop computer screen to which a conference microphone has been attached, participants were presented with 60 sentences, divided over three series of 20 sentences. Mini breaks marked the transitions between the series (signaled by means of a blank screen) and were followed by two random text comprehension questions based on the sentences that had just appeared as part of that series. The 20 sentences within each series were presented in blocks of two, three, four, five, or six sentences, in a pseudo-randomized order: no two sets of the same length were presented adjacently and the final set was never one of six sentences. Prior to the actual test, participants received oral instruction, which was simultaneously presented in written form, and were given two trial sentences. Afterwards, the sentences appeared one-by-one and were continuously preceded by a fixation point (a cross), which appeared on the screen for a duration of 1000 ms. The sentences were projected for a maximum duration of 6000 ms or upon pressing the spacebar. At the end of each set, the word RECALL (HERINNERING in the Dutch version) was projected on the screen, encouraging subjects to recall (freely) as many sentence-final words as possible. These were recorded (in the order in which they were named by the participant) on a set scoring form. A score of 1 was subsequently awarded to each correct word. While recorded, the order of recall did not influence the scores In line with [37], the Reading Span score constituted all correct items added together. The maximum Reading Span score was therefore 60 (the number of sentences in the test).

#### 3.2.3. Stroop Task

As was the case for the Reading Span, the Stroop test was administered twice: once for Dutch and once for English. This means that during the first test subjects were instructed to respond to the ink colors in Dutch and in the second in English. Three colors were used in the Stroop test: yellow, black and pink. These colors were picked because they are among the few color words that are not phonologically similar in Dutch and English (the Dutch color words are geel, zwart, and roze, respectively) (see [38]). 

In line with the Reading Span Task, the Stroop paradigm was programmed in Zep experimental software, run under Linux Ubuntu. Participants were again positioned as a distance of 60 cm. From a white laptop screen to which a three-button response box had been attached. Following a 1000 ms fixation point, the color words were projected one-by-one in the middle of the screen. Subjects were asked to press the response box buttons that had been taped with yellow, black, and pink to correspond to the color words. All color words were displayed in a set order until subjects pressed the key, with a maximum duration of 4000 ms, following [6]. Each response automatically led to the next fixation point. All sessions were preceded by a block of six trial items. In the two blocks (one for Dutch and one for English) 12 trials for each color were presented (six congruent ones and six incongruent ones). Because of the three colors that were used in this design, that resulted in a total of 36 items. In addition to the 36 items, 14 neutral trials were added. These neutral trials were words presented in an unfamiliar language for all participants, namely Georgian (familiarity with foreign languages other than L2 English had been checked by means of a screening questionnaire). In this study a manual rather than oral Stroop was employed for several reasons. Although manually responding to colors can perhaps be seen as an unnatural response, it does ensure that no data are lost due to participants not speaking loud enough or technical failure of the recording equipment. In addition, it has been argued that, with training (achieved through a sufficient number of trial items in this study), manual Stroop tests work rather well [39]. The number of correct responses was recorded, but more importantly for the present purposes, the Stroop effect was computed for each language by subtracting the reaction time on the congruent items from that of the incongruent items. The smaller the value, the better the inhibitory control. 

## 4. Results

To gain more insights into the language proficiency of the participant pool, two C-tests were administered: one for L2 English and one for L1 Dutch. Table 2 below shows the mean scores and standard deviations on both these measures, split per age category:

**Table 2:** Mean scores (and SDs) on the English and Dutch C-test, split per age category.

	40–50 (*n* = 16)	60–70 (*n* = 17)	71+ (*n* = 29)
L2 English C-test (max = 100)	75.81 ± 13.05 Range: 45–92	78.88 ± 10.98 Range: 57–93	68.76 ± 17.46 Range: −15-95
L1 Dutch C-test (max = 100)	92.25 ± 5.41 Range: 79–100	86.94 ± 9.67 Range: 69–98	73.04 ± 23.91 Range: −21-97

As can be seen from Table 1, the same pattern emerged for both languages across the three age groups: all participants obtained higher scores for L1 Dutch than L2 English, with the score on the Dutch C-test significantly outweighing that on the English equivalent for the youngest two age categories (assessed by means of a paired-samples *t*-test): *t*(15) = −4.825, *p* < 0.000 for the 40–50 group; *t*(16) = −3.343, *p* < 0.005 for the 60–70 participants. It is furthermore interesting to point out that the C-test scores obtained from the Dutch monolingual control group—although slightly higher—did not differ significantly from the data obtained from the Dutch-English bilinguals, for none of the age groups. This suggests that when measured on overall language proficiency, the Dutch-English bilinguals did not display any L1 attrition effects (see [40] for more background on L1 attrition). Conversely, the English C-test scores obtained from the monolingual English control group were markedly higher than those of the bilinguals, suggested that the participants at all ages might have been Dutch-English bilinguals, but the vast majority was not balanced. For the group that had been in Australia the longest, *i.e.*, the 71+ group, no significant difference was found and they performed equally well in both languages. It should be noted, however, that this performance was markedly lower for both the L1 and L2 than that of their younger peers. One-way analyses of variance (ANOVAs) revealed the score differences between the groups to be significant for both L1 Dutch (*F*(2,55) = 7.688, *p* < 0.005) and L2 English (*F*(2,56) = 3.302, *p* < 0.05). Subsequent *post hoc* Tukey procedures showed that this effect in the English C-test was only near significant for the 60–70 *versus* 71+ groups (*p* = 0.051). However, for the Dutch version, the effect was significant for both the youngest age group *versus* the oldest one (*p* < 0.005) and the 60–70 year-olds *versus* the 71+ participants (*p* < 0.05). At no point did the younger two groups significantly differ in scores. This effect may be directly related to the difference in educational background, but only one correlation was found; the higher educated participants in the 71+ group generally did better on the English C-test (*r* = 0.415, *p* < 0.05). A similar link was not found for the Dutch C-test or any of the other age groups under investigation. The difference in scores more likely results from age-related language changes, such as difficulties in word retrieval (see Section 2.4.), which is essential for a task like the C-test. 

Table 3 below depicts the descriptives of the L1 Dutch and L2 English WM scores, obtained via the Reading Span paradigms. 

**Table 3:** Mean WM scores (and SDs) on the L1 and L2 Reading Span Tasks, split per age category.

	40–50 (*n* = 17)	60–70 (*n* = 17)	71+ (*n* = 29)
L2 English WM scores (max = 60)	40.53 ± 5.363 Range: 28–50	36.41 ± 5.269 Range: 28–46	30.03 ± 6.378 Range: 18–46
L1 Dutch WM scores (max = 60)	41.88 ± 3.855 Range: 34–48	37.35 ± 4.499 Range: 30–46	30.79 ± 7.360 Range: 14–51

In line with the L2 proficiency scores, and also conform the predictions formulated for the first research question, the L1 and L2 WM scores closely resembled each other and were, in fact, not statistically different for any of the three age groups. To examine the first research question more closely, separate bivariate correlation analyses were run for each age group, yielding a significantly positive correlation for the youngest group: the better an individual performed on the L2 proficiency test, the higher the L2 WM score: *r* = 0.534, *p* < 0.05. This same tendency was found for the 60–70 year-old participants, although only a near-significant value was reached here (*r* = 0.375, *p* = 0.059), but not for the oldest individuals. What was observed was an almost linear decline in WM scores, in line with the aging literature, where the groups differed significantly from each other (assessed by means of a one-way ANOVA) in both L1 WM scores (*F*(2,60) = 20.109, *p* < 0.000) and L2 WM scores (*F*(2,60) = 18.469, *p* < 0.000). For L1 WM scores, Tukey *post hoc* tests located this effect for the youngest *versus* the oldest participants (*p* < 0.000) as well as the 60–70 group and the 71+-ers (*p* < 0.005), but also bordering on significance for the participants aged 40–50 years *versus* those in their 60–70s: *p* = 0.072. This same tendency was observed for L2 WM scores: 40–50 year-old participants did better than the 71+ year-olds (*p* < 0.000), but did not differ from the 60–70 year-old group, who in turn outperformed their older peers (*p* < 0.005). Whereas the bilingual participants in this study were asked to complete the reading span test in both Dutch and English, it is interesting to note that their performance on both versions was statistically similar to that of the monolingual Dutch control group (with respect to the Dutch Reading Span) and the Anglophone control group (whom were asked to complete the English Reading Span). To answer the second research question, whether L1 WM scores are predictive of L2 WM scores and whether this relation depends on age, three separate bivariate (linear) regression analyses were carried out (one for each age category), using L1 WM and L2 C-test scores as the independent variables and L2 M scores as the dependent variable. The regression model that was constructed for the 40–50 group accounted for 31.7% of the total variation in scores within this group. Crucially, it was not the L1 WM score (β = 0.178, *t*(16) = 0.774, *p* = 0.453), but the L2 proficiency score that significantly predicted an individual’s L2 WM score (β = 0.515, *t*(16) = 2.234, *p* < 0.05). In the participants aged 60–70, more of the variation in scores could be accounted for by the regression model: 47.5%. Contrary to their younger peers, however, it was not L2 proficiency, but L1 WM scores that predicted L2 WM scores: β = −0.118, *t*(16) = 0.611, *p* = 0.551 for the C-test and β = 0.227, *t*(16) = 3.525, *p* < 0.005 for the L1 WM scores. This same tendency was found for the oldest participants in this study, but even more of the variations in scores within this group could be accounted for by means of the regression model (65.9%). Here, also, the L1 WM score was a significant predictor of L2 WM score: β = 0.765, *t*(28) = 5.915, *p* < 0.000, but L2 proficiency was not: β = 0.116, *t*(28) = 0.9894, *p* = 0.381. In sum, the tendencies found were the same for the oldest subjects who had been in their L2 environment longest, as compared to their younger peers, with a much shorter length of residence in Australia. 

Table 4 can shed more light on the third research question, whether language proficiency was related to the ability to inhibit interfering linguistic information. The table first of all lists the L1 and L2 Stroop scores for all three age groups. Not only the mean reaction times on the congruent trials are provided, but also those for the incongruent and neutral trials. In addition, the Stroop effect scores (arrived at by subtracting an individual’s mean reaction time on the congruent Stroop items from the incongruent ones) are reported, as well as the accuracy scores. As prior descriptive analyses had not revealed clear outliers, no data cleansing took place. 

**Table 4:** Mean reaction times (and SDs) on the L1 and L2 Stroop Tasks, split per age category.

		40–50 (*n* = 17)	60–70 (*n* = 16)	71+ (*n* = 27)
Accuracy score (max = 50)	Dutch	47.50 ± 6.41	47.47 ± 3.09	41.17 ± 13.50
	English	49.65 ± 0.79	49.19 ± 1.22	46.56 ± 5.69
RTs congruent trials	Dutch	800.63 ± 84.07	966.47 ± 218.59	1147. 97 ± 555.55
	English	662.59 ± 140.63	752.89 ± 150.77	958.12 ± 324.74
RTs incongruent trials	Dutch	851.23 ± 187.45	1125.53 ± 239.20	1305.17 ± 546.22
	English	707.55 ± 150.40	837.59 ± 159.05	1139.32 ± 402.51
RTs neutral trials	Dutch	783.42 ± 173.69	1011.38 ± 218.28	1271.86 ± 562.76
	English	680.53 ± 150.88	755.73 ± 170.84	1038.87 ± 434.86
Stroop effect	Dutch	50.60 ± 93.57	159.06 ± 144.02	157.20 ± 292.41
	English	44.95 ± 44.95	84.71 ± 80.50	181.20 ± 140.76

Note: The units in Table 4 represent milliseconds.

Interesting trends can be distilled from these scores. First of all, the bilinguals displayed an almost linear increase in reaction time as a function of age (assessed by means of a one-way ANOVA test of variance), for congruent items, congruent items as well as neutral items, as expected. This effect was replicated in both monolingual control groups. In the bilingual group, this change was found for both the Dutch and English Stroop test, although different patterns emerged for both languages. In the Dutch test, no significant differences across age groups were found for accuracy scores, nor for the Stroop effect itself. However, the 40–50 year olds were significantly faster at responding to congruent items in comparison to the oldest group of participants (*F*(2,57) = 3.721, *p* < 0.05) and this was also found for the incongruent and neutral trials *F*(2,57) = 6.265, *p* < 0.005 and *F*(2,57) = 7.262, *p* < 0.005, respectively). The 60–70 year-olds were not found to be either significantly slower than the middle-agers, or faster than their oldest old peers. Whereas in the Dutch Stroop age-related differences were not attested in relation to accuracy scores and the Stroop effect, all aspects under investigation yielded significant differences across the bilingual age groups in the English version, illustrated in Table 5.

**Table 5:** ANOVA test statistics in relation to the English Stroop test with age as factor.

English Stroop test	ANOVA test statistics
Accuracy scores	*F*(2,57) = 4.037, *p* < 0.05
RTs congruent items	*F*(2,57) = 8.454, *p* < 0.001
RTs incongruent items	*F*(2,57) = 12.418, *p* < 0.000
RTs neutral items	*F*(2,57) = 7.90, *p* < 0.001
Stroop effect	*F*(2,57) = 9.558, *p* < 0.000

Follow-up Tukey *post hoc* texts revealed that the youngest subjects consistently outperformed the oldest old participants: *p* < 0.05 for accuracy; *p* < 0.005 for neutral trials; *p* < 0.001 for congruent trials; and *p* < 0.000 for both incongruent trials and the Stroop effect. The youngest and youngest old age groups did not differ from each other at any point, but the youngest old subjects did do better than their oldest old counterparts regarding congruent and neutral trials as well as the Stroop effect (*p* < 0.05 in all cases) and on incongruent trials (*p* < 0.01). 

To look more closely at the bilinguals’ potentially different performance on the Dutch *versus* English Stroop, paired-samples *t*-tests were administered on the basis of accuracy, congruent, incongruent and neutral items, and Stroop effect scores. As can be seen from Table 4, the participants almost invariably obtained higher accuracy scores, responded faster, and showed smaller Stroop effects when the Stroop was administered in English, their L2. While this discrepancy was not significant in the case of the Stroop effect, it was in nearly all other cases, reflected in Table 6 below.

**Table 6:** Paired-samples *t*-test statistics for the Dutch *versus* English Stroop test.

Bilinguals’ performance on the Dutch *versus* English Stroop test	Age groups		
	40–50	60–70	71+
Accuracy	n.s.	*t*(14) = −2.310, *p* < 0.05	*t*(26) = −2.419, *p* < 0.05
RTs congruent trials	*t*(15) = 4.649, *p* < 0.000	*t*(14) = 3.888, *p* < 0.005	n.s.
RTs incongruent trials	*t*(15) = 4.654, *p* < 0.000	*t*(14) = 4.861, *p* < 0.000	n.s.
RTs neutral trials	*t*(15) = 3.234, *p* < 0.01	*t*(14) = 5.205, *p* < 0.000	*t*(26) = 3.126, *p* < 0.005
Stroop effect	n.s.	n.s.	n.s.

The performance of the bilinguals was, finally, set off against that of the monolingual Dutch speakers (in relation to the Dutch Stroop) and monolingual English speakers (the English Stroop test) by means of independent samples *t*-tests, run separately for all age groups. Apart from one instance where the 40–50 year-olds showed significantly slower response latencies on the neutral trials compared to their monolingual peers, the Dutch Stroop yielded no significant difference at all between the bilinguals and monolingual Dutch speakers. In the English version, by contrast, there were quite a number of significant differences between the bilinguals and the monolingual English speakers. In all cases, the bilinguals outperformed the monolinguals: they obtained higher accuracy scores, smaller Stroop effects and faster response latencies for congruent, incongruent, and neutral items (see Table 7). 

**Table 7:** Independent samples *t*-test statistics for the bilinguals *versus* English monolinguals.

Bilinguals’ *versus* monolingual English speakers’ performance on the English Stroop Test		Age groups	
	40–50	60–70	71+
Accuracy	*t*(21.523) = 3.497, *p* < 0.005	*t*(26.012) = 2.394, *p* < 0.05	n.s.
RTs congruent trials	*t*(29.837) = −3.524, *p* < 0.001	*t*(31.824) = −4.066, *p* < 0.000	*t*(22.311) = −2.940, *p* < 0.01
RTs incongruent trials	*t*(29.523) = −4.314, *p* < 0.000	*t*(32.728) = −4.483, *p* < 0.000	*t*(40) = −2.837, *p* < 0.01
RTs neutral trials	*t*(26.886) = −3.679, *p* < 0.001	*t*(34.552) = −4.238, *p* < 0.000	*t*(40) = −2.441, *p* < 0.05
Stroop effect	*t*(23.499) = −2.601, *p* < 0.05	n.s.	n.s.

The fourth research question examined the interaction between WM capacity and inhibitory control and the role of age and/or L1 and L2 proficiency. In order to investigate this, separate bivariate correlation analyses for each age group were carried out, initially focusing on the relationship between L1 WM scores and L1 Stroop results and L2 WM and L2 Stroop scores. These analyses were followed by correlation analyses on the basis of L2 Stroop scores and L1 WM scores, separately repeated for all age groups under investigation, Starting with the L1, no significant correlation was found in the bilinguals—for any age group under investigation—between L1 WM scores and L1 Stroop effect outcomes, which was similar to the outcome of the Dutch monolinguals. In other words, there appeared to be no relation whatsoever between an individual’s working memory capacity (measured in the L1) and ability to inhibit (L1) linguistic information. The same outcome emerged for the L2: the bilinguals—of all ages—displayed no significant relationship between L2 WM scores and L2 Stroop performance. While the same result was replicated for the younger monolingual English speaker, it is remarkable that only the oldest L1 English speakers showed the expected inverse relationship between L1 WM scores and L1 Stroop scores (*r* = −0.543, *p* < 0.05). Finally, and similar to the L1 and L2 analyses separately, no correlation was found between the bilinguals’ L1 WM scores and how well they were able to inhibit linguistic information in their L2 and this same tendency was found for all ages.

## 5. Discussion

This study was first of all able to classify the participants under investigation as near-native L2 English speakers and, more accurately, bilinguals. This in turn impacted on the results. Indeed, although the two groups comprising both the 40–50 year-old participants and those aged between 60 and 70 did prove to be Dutch-dominant (on their basis of their significantly higher scores for the Dutch C-test compared to the English equivalent), no such significant difference was found for the oldest, 71+, participants. The factor length of residence in the L2 environment was, in relation to this, found to correlate with L2 proficiency for this latter group, but not for their younger peers. This very much seems to suggest that the participants who had been in their L2 environment longest were the most balanced bilinguals. Having said that, the 71+-ers’ scores on the C-test were significantly lower (for both languages) than those of their younger peers. This is partly explained by the number of years of formal education, reflecting the changing demographics of older *versus* more recent Dutch immigrants to Australia; whereas in the years directly following World War II most people moved for economic reasons, most recent immigrants are young (highly-educated) professionals who do not refer to themselves as immigrants but, rather, prefer the term expat [41]. At the same time, this drop in scores can also be explained as an aging effect: lexical access difficulties frequently reported for older adults may have led to lower scores on the C-test. 

The lower scores produced by the older adults extends beyond the C-test and was in fact a trend that was discernible throughout the data: the participants aged 71 or older were found to have smaller working memory capacities as well as reduced inhibitory control mechanisms than their “youngest old” peers, who in turn often scored significantly lower than the middle-aged group, in line with what is known about aging processes [8,11,16]. The overall role of L2 proficiency was less clear from the data, often only revealing effects for the least proficient 40–50 group, whose stay in the L2 setting had been considerably shorter than that of the older adults.

To focus on the research questions and predictions in more detail and starting with the first research question, L2 proficiency was indeed found to relate to L2 WM scores in the sense that all participants under investigation proved (on the basis of their L2 C-test scores) to be highly proficient in English. This, in turn, led to their L1 and L2 WM scores being statistically similar. Purely based on the bivariate correlation analyses that were run, however, the C-test scores only significantly correlated with the scores on the L2 Reading Span Task for the 40–50 year-olds and not for the older groups. This can perhaps be explained on the basis of individual variation. In fact, the length of residence as well as L2 proficiency scores varied considerably in the youngest group, possibly causing the discrepancy. In line with the predictions, there was a linear decrease in WM scores, for both languages, as a function of age. Apart from the frequently reported WM capacity decline in older adults, for the Reading Span Task (as opposed to the more frequently administered digit spans) the effect could also be due to lexical access being impaired in older adults, making it hard to access the sentence-final words they need to recall. 

The second research question was directly related to the first and examined whether L2 WM scores could be predicted on the basis of L1 WM scores and how this relationship varied as a function of L2 proficiency and/or age. A clear link between L1 and L2 WM scores was found, but here too a trend appeared not unlike the one seen for the first research question: whereas the score on the L2 Reading Span Task was mostly dependent on L2 proficiency (as opposed to L1 WM) for the youngest age group, it was L1 WM capacity that was the most important predictor of L2 WM scores for both older groups. It is likely that L2 proficiency did not predict L2 WM for the two oldest groups—and notably the oldest group—as they had already proven to be the most proficient on the English C-test. This again ties in with length of residence, but although a positive correlation between L2 WM and length of residence was found for the oldest group, no such link could be established for the youngest old or middle-aged group. 

As part of the third research question, the prediction was that the response latencies and the Stroop effect scores in both the L1 and L2 would be relatively large for all groups (given their high L2 proficiency levels), but that it would be largest still in relation to the L1 (although perhaps not significantly so). In addition, the oldest group was expected to be slower overall and also show the largest error rate. Not all of these predictions were borne out of the data. The scores for all groups were relatively large on both the L1 and L2, suggestive of language processing occurring in both languages, as opposed to what was found for the beginning learners of L2 Spanish in [6]. The response latencies increased incrementally with advanced age, and at the same time a decrease in accuracy scores was observed in older adults. Furthermore, more interference was attested in the L1 (evidenced by larger response latencies and lower accuracy rates in the L1 Dutch Stroop). However, contrary to expectation, this discrepancy was often significant. This does reveal the unbalanced nature of the bilinguals’ language proficiency. What is furthermore interesting, and no predictions of this kind had been formulated beforehand, is that the bilinguals tended to outperform the monolinguals and while this was not uniformly observed in relation to the Dutch monolinguals, this effect was very strong with respect to the English natives. This outcome strongly points in the direction of a bilingual cognitive control advantage (*cf.* [42]). This is underscored by the non-significant discrepancy between the bilinguals and monolingual Dutch speakers, the latter of which are at least receptively bilingual (see Section 3). What is clear is that the participants in this study processed both L1 and L2 incongruent Stroop items as interfering stimuli, which was not found for the less proficient L2 speakers in [6]. 

Perhaps the most puzzling finding, because it does not relate to the earlier findings in [6], is the one obtained for the fourth research question. Whereas an inverse relation was expected between WM and Stroop outcomes, no correlation of any nature was found here, not for any of the groups under investigation (the one exception to this being the oldest age group of the Australian controls). This result is not easily explained. A perhaps far-fetched explanation could be that, for this particular population, the constructs of working memory capacity and inhibitory control are in fact separable rather than tapping into one underlying executive control construct (see [8] and also the discussion in Section 2.2.), but that is extremely speculative at this point and more work is needed in order to shed more light on this idea.

## 6. Conclusions

This study has revealed clear trends for the (bilingual) L1 Dutch speakers of advanced L2 English under investigation on measures of language proficiency as well as WM capacity and inhibitory control. On the language measures and particularly L2 proficiency, the oldest groups (60–70 and 71+) clustered together in that they had generally attained a better command of L2 English relative to their L1 Dutch proficiency. The cognitive measures, on the other hand (*i.e.*, the Reading Span and Stroop Tasks) revealed a closer resemblance between the youngest two groups as opposed to the oldest group; the 71+ participants performed significantly lower on both WM capacity and inhibitory control. 

One of the outstanding questions that [6] formulated on the basis of their study was how L1 and L2 WM capacity and L1 and L2 inhibition are related to L2 proficiency in learners who have obtained a very good, perhaps even native-like command, of their L2. This study has indeed shown a much more intricate system of interrelatedness for such advanced learners, but in addition has shown that these dependencies may change across the lifespan and that aging, thus, plays an important role. That is not to say that no questions remain. The lack of correlations, especially of any kind for WM capacity and inhibition scores, cannot readily be explained on the basis of the data and more work is needed in this respect.

At the same time, it would be interesting for future work to take up the issue of the cut-off point regarding L2 proficiency after which incongruent L2 Stroop items are processed as interfering stimuli. To do this, a middle ground needs to be established between the relatively low-proficient L2 learners of Spanish in [6], and the near-native speakers in this study. A good avenue would be advanced L2 speakers, enrolled in bilingual education, for instance, or university majors, who are not immersed in their L2 environment the way the participants in this study were. In a previous study involving L1 Dutch learners of L2 English, both at high-school and university level, participants were found to be surprisingly insensitive to English spelling patterns [43], but how the L2 is processed and the role of WM and especially inhibition in this process remains a largely uncharted territory. 

Such a growing body of literature on the role of individual (cognitive) factors involved in language learning could even provide a blueprint of the successful (late) L2 language learner. Moving away from the previously dominant focus on motivation as a predictor for individual differences in L2 success, language learners are perhaps best classified in their ability to allocate their WM resources as well as more general executive control functions to the L2 learning task at hand. 
